# Neoadjuvant immunotherapy: new horizon for lymph node preservation

**DOI:** 10.1002/mco2.577

**Published:** 2024-05-13

**Authors:** Nian‐Nian Zhong, Bing Liu, Lin‐Lin Bu

**Affiliations:** ^1^ State Key Laboratory of Oral & Maxillofacial Reconstruction and Regeneration, Key Laboratory of Oral Biomedicine Ministry of Education, Hubei Key Laboratory of Stomatology School & Hospital of Stomatology, Wuhan University Wuhan China; ^2^ Department of Oral & Maxillofacial—Head Neck Oncology School & Hospital of Stomatology, Wuhan University Wuhan China

**Keywords:** cancer treatment, immunotherapy, lymph node, organ preservation, T cell, treatment de‐escalation

## Abstract

The study by Rahim et al., focusing on preoperative immunotherapy, highlights the pivotal role of CD8^+^ T cells within lymph nodes in response to neoadjuvant immunotherapy, suggesting that preserving lymph node integrity could bolster the treatment's efficacy by activating antitumor T cells. This underlines the importance of lymph node preservation and supports the use of immunotherapy as a neoadjuvant approach in cancer treatment.

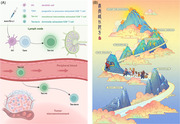

1

In a seminal study, Rahim et al.^1^ embarked on a clinical trial assessing preoperative immunotherapy using the programmed cell death ligand 1 (PD‐L1) inhibitor atezolizumab in patients diagnosed with locally metastatic head and neck squamous cell carcinoma (HNSCC). Their findings were disclosed in *Cell* in March 2023. Investigating the role of CD8^+^ T cells, the team collected diverse samples, including tumor tissues, uninvolved lymph nodes (uiLNs), metastatic lymph nodes (metLNs), and blood, and employed mass cytometry, single‐cell genomics, and multiplexed ion beam imaging for analysis.

The researchers identified that progenitor or precursor exhausted cells (Tpex) in uiLNs might be instrumental in modulating the human immune response to immunotherapy. Significantly, the decrease in Tpex and increase in transitional intermediate exhausted cells (Tex‐int), which can differentiate into terminally exhausted cells (Tex‐term), after immunotherapy, may be attributed to Tpex differentiation into Tex‐int (Figure 1A). These cells are predominantly found proximate to dendritic cells and display elevated PD‐1 expression, implying their activation and subsequent differentiation in response to the therapeutic intervention.

**FIGURE 1:**
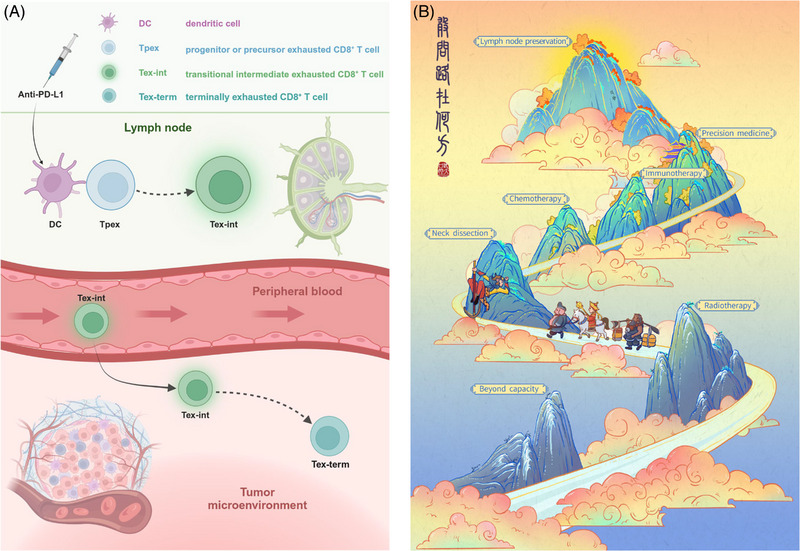
(A) After anti‐PD‐L1 neoadjuvant immunotherapy, Tpex within the lymph nodes differentiate into Tex‐int. These cells then enter the circulatory system, undergo proliferation, and subsequently infiltrate the tumor microenvironment. Created with BioRender.com. (B) The arduous journey of improving neck management in cancer therapy has been marked by significant advancements. Building on the foundational work of prior researchers, the field has entered a new era of cancer immunotherapy, brimming with potential. As we explore this innovative epoch, questions emerge about novel strategies for lymph node preservation in cancer therapy. The title of this illustration, “Where is the way forward?,” symbolizes the courage to confront these questions.

Interestingly, Tex‐int cells are discernible not only in uiLNs but also in the circulatory system, signaling their migration from lymph nodes (LNs) to the tumor milieu. Postimmunotherapy, there was a marked escalation in Tex‐int levels in the bloodstream, accompanied by a pronounced expression of the proliferation indicator, Ki‐67. On the other hand, metastatic activity seems to attenuate the immune response, as evidenced by diminished CD8^+^ T cell activity in the blood of patients harboring metLNs posttreatment, which hints at the existence of immunosuppressive cellular niches. Nonetheless, the therapeutic regimen exhibited efficacy in amplifying T‐cell responsiveness within metLNs.

Metastasis stands as a hallmark biological characteristic of cancers, with LN metastasis indicating a poor prognosis in the majority of solid tumors. The “PUMP” principle effectively encapsulates this process, dividing it into four stages: preparation, unleash, migration, and planting.^2^ Consequently, the optimal management of LN metastasis in oncology remains an ongoing challenge. For instance, the current dominant treatment paradigm for HNSCC revolves around surgery and follows a multidisciplinary sequential protocol, which could be termed the “PRECISE” model (Prevention, Radiology, preoperative Evaluation, Chemotherapy, Immunotherapy, Surgery, and postoperative Evaluation).^2^ Over the past two centuries, treatments for cervical LN metastasis have progressed from initial inefficacy to today's comprehensive approach that incorporates neck dissection, radiotherapy, chemotherapy, and immunotherapy (Figure 1B). This journey necessitates overcoming manifold challenges and relentlessly pursuing innovation. Based on cold yet clear clinical data, at present, neck dissection remains essential for patients with HNSCC. However, given the pronounced decline in patients’ quality of life due to the trauma of neck dissection, it prompts the critical inquiry: where exactly lies the path to LN preservation?

Neoadjuvant immunotherapy advances the timing of adjuvant immunotherapy to the preoperative phase. It is widely regarded as effective due to the activation of a broader array of tumor T cell clones and enhanced antitumor T cell responses, attributed to the extensive exposure to tumor antigens prior to surgery.^3^ However, the precise origin of these responsive T cells remains elusive; they might emerge from peripheral blood or tumor‐draining LNs (tdLNs). A study by Spitzer et al.^4^ suggests that immunotherapy primarily activates LNs rather than intratumoral T cells. While the predominant focus of immunotherapies is on rejuvenating fatigued T cells within tumors, bolstering the immune response within LNs could potentiate the therapeutic efficacy. Notably, peripheral solid tumors disseminate via regional lymphatic vessels and tdLNs, which exhibit an essential yet paradoxical role in cancer. Although LN removal is routine in cancer interventions, tdLNs are central to immune responses, given their intricate relationship with immune cells. Hence, surgeries that involve LN extraction could diminish the T cell responses within LNs, potentially compromising immunotherapy's impact.

This pioneering investigation conducted by Rahim et al. last year marked the first clinical trial to examine the impact of immunotherapy on T cells in LNs, illuminating their pivotal role in orchestrating dynamic immune responses. Administering immunotherapy prior to LN dissection enables researchers to effectively study how immunotherapy influences LN metastasis and the nodes’ reactions to the treatment. The findings advocate for the preservation of LN integrity prior to administering immunotherapy, as this may bolster its efficacy by stimulating antitumor T cells in adjacent nodes. Once activated, these T cells have the potential to circulate throughout the bloodstream, targeting malignant cells. These groundbreaking discoveries support the use of immunotherapy as a neoadjuvant intervention. Rather than considering neoadjuvant immunotherapy as merely an advance in the timing of immunotherapy, it is more aptly described as a strategy that delays LN dissection, which aims to preserve LNs for their role in combating tumors.

The goals of modern management for cancer patients can be encapsulated in the “3L” goals—living, live long, and live lively.^5^ Neoadjuvant immunotherapy holds promise for enhancing long‐term survival and quality of life in cancer patients. It also offers hope in reducing complications associated with LN dissection through a LN preservation strategy, even though its full realization remains a challenging goal. This is due to the lengthy journey from basic research to clinical application, which requires substantial evidence to support changes in cancer surgical practices. It is crucial to carefully consider potential risks, such as cancer recurrence, metastasis, or delays in receiving more aggressive treatments that are necessary for some patients.

First, an in‐depth comprehension of tdLNs’ role in immunotherapy is imperative, necessitating the refinement and expansion of immunotherapeutic strategies that harness the potential of these LNs. Second, given the nuanced immunological differences between metLNs and uiLNs, it becomes evident that additional research is required to devise accurate, noninvasive methodologies for discerning between the two. This would enable targeted strategies to enhance the immune response based on the specific characteristics of metLNs and uiLNs. A limitation of Rahim et al.’s study is its failure to analyze the mechanisms behind the decrease of Tpex in metLN and its implications for impaired responses to immunotherapy, presenting a promising direction for future research. Furthermore, future studies should pay more attention to the de‐escalation strategies brought about by neoadjuvant immunotherapy, whether targeting the primary tumor or the LNs. Expanding clinical trials to explore the feasibility of these strategies is essential for advancing our understanding and application of neoadjuvant immunotherapy in cancer management.

## AUTHOR CONTRIBUTIONS

*Writing—original draft, visualization*: Nian‐Nian Zhong. *Writing—review and editing, supervision*: Bing Liu. *Writing—review and editing, supervision, funding acquisition*: Lin‐Lin Bu. All authors have reviewed and approved the final version of this manuscript for publication. Each author agrees to be accountable for all aspects of the work in ensuring that questions related to the accuracy or integrity of any part of the work are appropriately investigated and resolved.

## CONFLICT OF INTEREST STATEMENT

The authors declare no conflict of interest.

## FUNDING INFORMATION

This study was supported by Fundamental Research Funds for the Central Universities (Wuhan University, Clinical Medicine + X) (2042024YXB017), Natural Science Foundation of Hubei Province (2023AFB665), and Medical Young Talents Program of Hubei Province to L.‐L. Bu.

## ETHICS STATEMENT AND CONSENT TO PARTICIPATE

Not applicable.

## CONSENT FOR PUBLICATION

Not applicable.

## Data Availability

Not applicable.
